# Camrelizumab plus albumin-bound paclitaxel and S-1 as first-line treatment for patients with human epidermal growth factor receptor 2-negative advanced gastric or gastroesophageal junction adenocarcinoma: a phase 2 trial

**DOI:** 10.3389/fimmu.2025.1634502

**Published:** 2026-01-02

**Authors:** Chen Wu, Shuai Li, Xinfang Hou

**Affiliations:** Department of Medical Oncology, The Affiliated Cancer Hospital of Zhengzhou University & Henan Cancer Hospital, Zhengzhou, China

**Keywords:** gastric or gastroesophageal junction adenocarcinoma, camrelizumab, albumin-bound paclitaxel, human epidermal growth factor receptor 2-negative, S-1

## Abstract

**Background:**

Current first-line treatment options for human epidermal growth factor receptor 2 (HER2)-negative advanced gastric or gastroesophageal junction (G/GEJ) adenocarcinoma have limited efficacy. This study aimed to evaluate the efficacy and safety of camrelizumab plus albumin-bound paclitaxel and S-1 as first-line treatment for this population.

**Methods:**

In this phase 2 trial (NCT04675866), patients received albumin-bound paclitaxel (125 mg/m^2^, days 1 and 8) and S-1 (40–60 mg per body surface area, twice daily on days 1-14) for 4–6 cycles of 21 days each. Camrelizumab (200 mg every 3 weeks) was concurrently initiated and continuously administered until disease progression, intolerable toxicity, or completion of 2-year treatment. The primary endpoint was objective response rate (ORR).

**Results:**

Between December 2020 and December 2024, 47 patients were enrolled. By the data cutoff date (January 6, 2025), the median follow-up duration was 16.7 months (range, 1.6-42.2 months). Among the 40 patients with evaluable efficacy, the ORR was 67.5% (95% CI: 52.3%-82.7%). The disease control rate was 95.0%. Median progression-free survival was 7.8 (95% CI: 6.2-9.4) months, and median overall survival was 23.8 (95% CI: 15.2-32.4) months. Grade 3–4 treatment-related adverse events(TRAEs) occurred in 18 patients (38.3%), with the most common being decreased neutrophil count (12 [25.5%]) and anemia (four [8.5%]).

**Conclusion:**

The combination of camrelizumab, albumin-bound paclitaxel, and S-1 as first-line treatment for patients with HER2-negative advanced G/GEJ adenocarcinoma showed promising efficacy and an acceptable safety profile. Randomized controlled clinical trials are required to further demonstrate its efficacy and optimal application scenario.

**Clinical Trial Registration:**

https://clinicaltrials.gov/study/NCT04675866?term=Hou%20Xinfang&rank=1, identifier NCT04675866.

## Introduction

Gastric cancer is one of the most frequently diagnosed malignancies worldwide, posing a significant threat to human health. It ranks fifth in incidence and fifth in mortality globally (1). Due to its asymptomatic nature in early stages, the majority of patients are diagnosed at advanced stage. Advanced gastric cancer is associated with poor prognosis, with a 5-year survival rate of less than 10% (2). Human epidermal growth factor receptor 2 (HER2)-negative subtype accounts for approximately 90% of advanced gastric cancers. For this subtype, the median survival with combination chemotherapy is about one year (3). In recent years, the advent of immune checkpoint inhibitors (ICIs) has transformed first-line treatment for advanced gastric or gastroesophageal junction (G/GEJ) cancer. Pivotal CheckMate 649 and KEYNOTE-859 trials have established the role of nivolumab or pembrolizumab combined with chemotherapy as first-line treatment for advanced G/GEJ cancer (4, 5). Other ICIs, including sugemalimab, sintilimab, and toripalimab, have also been approved for use in this setting in China (6–8). However, the chemotherapy regimens used in these trials were predominantly fluoropyrimidine and platinum-based (9). Currently, there are significant regional differences in the selection of treatment regimens. In Europe and the Americas, docetaxel (as part of the FLOT regimen) has become the standard neoadjuvant chemotherapy for locally advanced gastric cancer (10). In all versions of the NCCN Guidelines, taxane-based drugs are also recommended as part of the “other recommended regimens” for first-line treatment (11). However, a single chemotherapy regimen is far from meeting the needs of clinical practice. Moreover, taxane-based drugs can exert a synergistic effect with PD-1 monoclonal antibody inhibitors to enhance efficacy (12–15). The search for more optimal immunochemotherapy combinations remains an important research direction.

Camrelizumab is a humanized high-affinity monoclonal antibody targeting programmed cell death-1 (PD-1) (16), which has been approved for use across multiple cancer types. A phase 2 trial suggested that camrelizumab combined with apatinib and chemotherapy achieved an impressive objective response rate (ORR) of 76.5% and a median progression-free survival (PFS) of 8.4 months for the first-line treatment of advanced gastric cancer, with a manageable safety profile (17). Similarly, another phase 2 trial of camrelizumab in combination with apatinib and S-1 for the second-line treatment of advanced gastric cancer reported promising efficacy and safety outcomes (18).

Albumin-bound paclitaxel is a solvent-free, albumin-stabilized formulation of paclitaxel colloid. This formulation offers a potential solution for more effective and tolerable treatment regimens (19). Studies have shown that albumin-bound paclitaxel can synergize with immunotherapy. Its albumin-bound formulation facilitates uptake by both tumor and immune cells, with internalized albumin-bound paclitaxel exhibiting marked immunostimulatory activity, thereby promoting cancer immunity cycles (12). Albumin-bound paclitaxel has been approved for the treatment of breast cancer (20), non-small cell lung cancer (21), and pancreatic cancer (22), demonstrating good anti-tumor activity. The Japanese phase 3 ABSOLUTE trial revealed that the median overall survival (OS) with albumin-bound paclitaxel was non-inferior to solvent-based paclitaxel, leading to its approval in Japan for the second-line treatment of advanced gastric cancer (23). In a neoadjuvant trial evaluating camrelizumab combined with albumin-bound paclitaxel and S-1, this regimen significantly improved tumor regression grade and pathological complete response rate in gastric cancer compared to the SOX (S-1 plus oxaliplatin) regimen, without increasing treatment-related adverse events (TRAEs) (10). Additionally, a meta-analysis demonstrated that albumin-bound paclitaxel provided better disease control rate (DCR) and prolonged survival in metastatic gastric cancer, with a manageable safety profile (24).

Thus, we conducted a clinical trial to evaluate the efficacy and safety of the combination of camrelizumab, albumin-bound paclitaxel and S-1 as first-line treatment for patients with advanced G/GEJ adenocarcinoma.

## Methods

### Study design and participants

This was a single-arm, single-center phase 2 trial, conducted at Henan Cancer Hospital in China. The inclusion criteria were: age of 18-75 years; pathologically confirmed HER2-negative G/GEJ adenocarcinoma; no previous history of systemic therapy for unresectable locally advanced or metastatic disease; any previous neoadjuvant or adjuvant therapy that had been completed at least 6 months prior to enrollment; Eastern Cooperative Oncology Group (ECOG) performance status of 0-2; at least one measurable lesion according to the Response Evaluation Criteria In Solid Tumors version 1.1. The main exclusion criteria were: previous treatment with albumin-bound paclitaxel or paclitaxel; history of other malignancies within the past 5 years (except adequately treated basal or squamous cell skin carcinoma, or *in situ* carcinomas such as cervical or breast carcinoma); presence of any uncontrolled serious medical condition that, in the judgment of the investigator, would compromise the participant’s ability to undergo the study treatment; participation in other interventional clinical trials within 28 days prior to enrollment, unless in the follow-up phase only.

The study was conducted in accordance with the Declaration of Helsinki. The study protocol was approved by the Ethics Committee of Henan Cancer Hospital (Ethics Approval number: 2020233-003) and registered with ClinicalTrials.gov (NCT04675866). All patients voluntarily consented to take part in the study and signed an informed consent form.

### Treatment

After enrollment, patients received combination therapy consisting of camrelizumab, albumin-bound paclitaxel, and S-1. Albumin-bound paclitaxel was administered intravenously at 125 mg/m² on days 1 and 8 of each 21-day cycle, and S-1 was administered orally based on body surface area (BSA; 40 mg per dose for patients with BSA <1.25 m^2^, 50 mg for BSA ≥1.25 m^2^ and <1.5 m^2^, and 60 mg for BSA ≥1.5 m^2^) twice daily on days 1–14 of each cycle. Chemotherapy was given for 4–6 cycles. Intravenous camrelizumab (200 mg every 3 weeks) was initiated concurrently with chemotherapy and continued as maintenance monotherapy for patients who had no disease progression after the completion of chemotherapy until disease progression, unacceptable toxicity, withdrawal of informed consent, or a determination by the investigator that discontinuation of treatment was necessary.

### Endpoints and assessments

The primary endpoint was ORR, defined as the proportion of patients with the best response of complete response (CR) or partial response (PR). Secondary endpoints were DCR (defined as the proportion of patients with the best response of CR, PR, or stable disease [SD]), PFS (defined as the time from enrollment to disease progression or any-cause death), OS (defined as the time from enrollment to any-cause death), and safety. The exploratory endpoint was the association of programmed cell death-ligand 1 (PD-L1) expression with efficacy. PD-L1 was evaluated using the combined positive score (CPS), defined as the total number of PD-L1-stained cells (including tumor cells, macrophages, and lymphocytes) divided by the total number of viable tumor cells observed microscopically, multiplied by 100 (25).

Tumor response was assessed according to RECIST 1.1 every 6 weeks for the first 6 months and every 3 months thereafter. Adverse events were graded according to the National Cancer Institute Common Terminology Criteria for Adverse Events version 5.0.

### Statistical analysis

The study used Simon’s two-stage design to determine sample size, with a one-sided significance level set at 0.05 and statistical power set of 80%. This study was designed in 2020, when immunotherapy had not yet been incorporated into clinical guidelines. At that time, the mainstream first-line treatment still mainly relied on combination chemotherapy. A meta-analysis of first-line paclitaxel combined with S-1 showed that the average objective response rate (ORR) was approximately 40.8% (26). In Cohort 2 of the KEYNOTE-059 study, Pembrolizumab combined with cisplatin and 5-fluorouracil was used for first-line treatment of advanced gastric/gastroesophageal junction (G/GEJ) cancer. The results showed an objective response rate (ORR) of 60% (27). At the 2019 American Society of Clinical Oncology (ASCO) Annual Meeting, Professor Shen presented the preliminary study data of a first-line treatment regimen for gastric cancer: carboplatin combined with capecitabine and oxaliplatin for 4–6 cycles, followed by sequential treatment with carboplatin combined with apatinib. The objective response rate (ORR) of this regimen was 69.2%. We assumed that the ORR could increase from 45% to 65%. In the first stage, 15 patients will be enrolled. If ≥7 patients achieve a partial response (PR) or complete response (CR), the second stage will proceed with the enrollment of an additional 24 patients. Accounting for a dropout rate of 10%, the total sample size was calculated to be 46 patients.

Efficacy was analyzed in the efficacy analysis set, including patients with evaluable results of imaging examinations. Safety was analyzed in the safety analysis set, including all patients with at least one cycle of study treatment. The confidence intervals (CIs) of ORR was estimated using the Clopper-Pearson method. Median values for PFS and OS, along with their 95% CIs, were estimated using the Kaplan-Meier method. Univariate Cox regression analysis was conducted to identify potential predictive factors influencing PFS and OS. All statistical analyses and visualization were performed using SPSS 27.0 and PRISM 9.0. A significance level of 0.05 was applied for all tests.

## Results

### Patient characteristics

**Figure 1:**
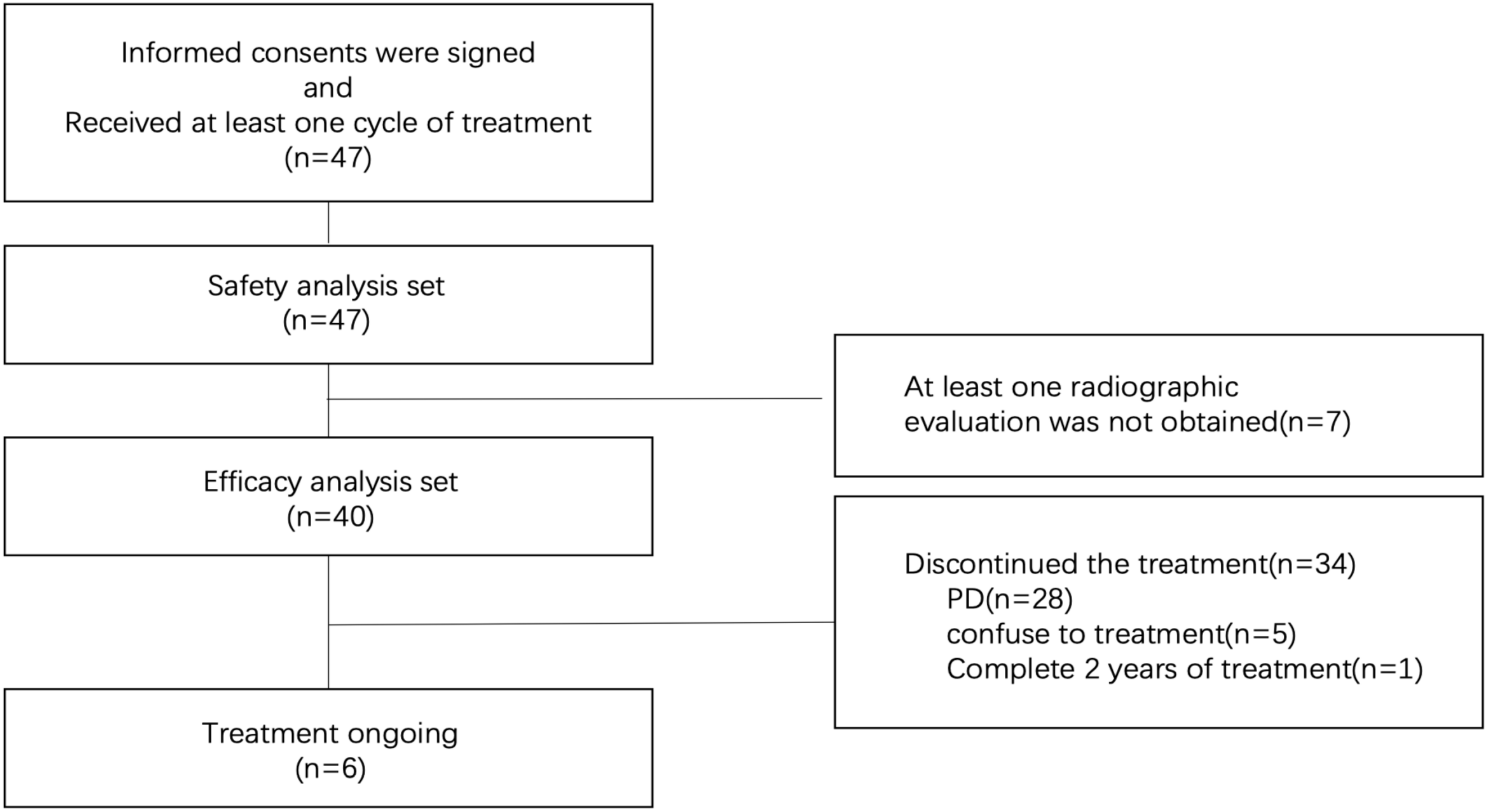
Patient flowchart. A total of 47 patients signed the informed consent forms and received at least one cycle of treatment, thus being eligible for the safety analysis. Seven patients failed to obtain at least one set of radiographic data due to regional restrictions caused by COVID-19, leaving 40 patients eligible for the efficacy evaluation. As of January 6, 2025, 6 patients were still continuing treatment in this study, while 34 patients had discontinued the study treatment. Among these 34 patients who discontinued treatment, 28 did so due to disease progression (PD), 5 refused further treatment, and 1 completed 2 years of maintenance treatment.

Between December 2020 and December 2024, a total of 47 patients were enrolled and received at least one cycle of study treatment (safety analysis set). Considering the potential for increased withdrawal due to the pandemic of coronavirus disease 2019 (COVID-19) pandemic, 47 patients were enrolled instead of the target 46. Due to regional restrictions imposed due to the COVID-19, seven had no available imaging results, while 40 were included in the efficacy analysis set (**Figure 1**). Of 47 patients, the median age was 57 years (range, 35–75 years), and 34 (72.3%) were male. All patients had stage IV disease, and seven (14.9%) had ECOG performance status of 2. The majority of patients (93.6%) had microsatellite-stable (MSS) and mismatch repair-proficient (pMMR) disease. The PD-L1 expression was detected by immunohistochemistry assays (Dako 22C3) in the central laboratory. Forty-six (97.9%) patients had available results of PD-L1 expression, including eight (17.0%) patients had PD-L1 ≥10, 14 (29.8%) had PD-L1 ≥5, and 28 (59.6%) had PD-L1≥1 (**Table 1**).

**Table 1:** Baseline characteristics.

Characteristic	Patients (N = 47)
Age (years), median (range)	57 (35-75)
<57	15 (31.9)
≥57	32 (68.1)
Sex	
Female	13 (27.7)
Male	34 (72.3)
ECOG performance status	
0	20 (42.6)
1	20 (42.6)
2	7 (14.9)
Histology	
Adenocarcinoma	38 (80.9)
Signet-ring cell carcinoma	9 (19.1)
PD-L1 expression(CPS 22c3)	
<1	18 (38.3)
≥1 and <5	14 (29.8)
≥5 and <10	6 (12.8)
≥10	8 (17.0)
Unknown	1 (2.1)
EBER	
Negative	42 (89.4)
Unknown	5 (10.6)
HER2 status	
IHC 0	20 (42.6)
IHC 1+	15 (31.9)
IHC 2+/FISH-	12 (25.5)
MSI/MMR status	
MSI/dMMR	1 (2.1)
MSS/pMMR	44 (93.6)
Unknown	2 (4.3)
Peritoneal metastases	
Yes	38 (80.9)
No	9 (19.1)
Bone metastases	
Yes	10 (21.3)
No	37 (78.7)
Number of metastatic sites	
1	16 (34.0)
2	18 (38.3)
3	8(17.0)
≥4	5 (10.6)

Data are n (%), unless otherwise indicated. ECOG, Eastern Cooperative Oncology Group; PD-L1, programmed cell death-ligand 1; EBER, Epstein-Barr virus-encoded RNA; HER2, human epidermal growth factor receptor 2; IHC, immunohistochemistry; FISH, fluorescence *in situ* hybridization; MSI, microsatellite instability; MMR, mismatch repair; dMMR, mismatch repair-deficient; MSS, microsatellite-stable; pMMR, mismatch repair-proficient.

By the data cutoff date of January 6, 2025, the median follow-up duration was 16.7 (range, 1.6-42.2) months, six patients were still on treatment, Twenty-eight patients experienced disease progression, 5 patients refused further treatment, and 1 patient completed 2 years of maintenance therapy. The median duration of treatment was 7.17 months (95% confidence interval [CI], 5.69n (14.9%) had e intervatients with disease progression, 16 subsequently received irinotecan-based chemoimmunotherapy, 7 received oxaliplatin-based chemoimmunotherapy, and 1 received apatinib treatment.

### Efficacy

**Figure 2:**
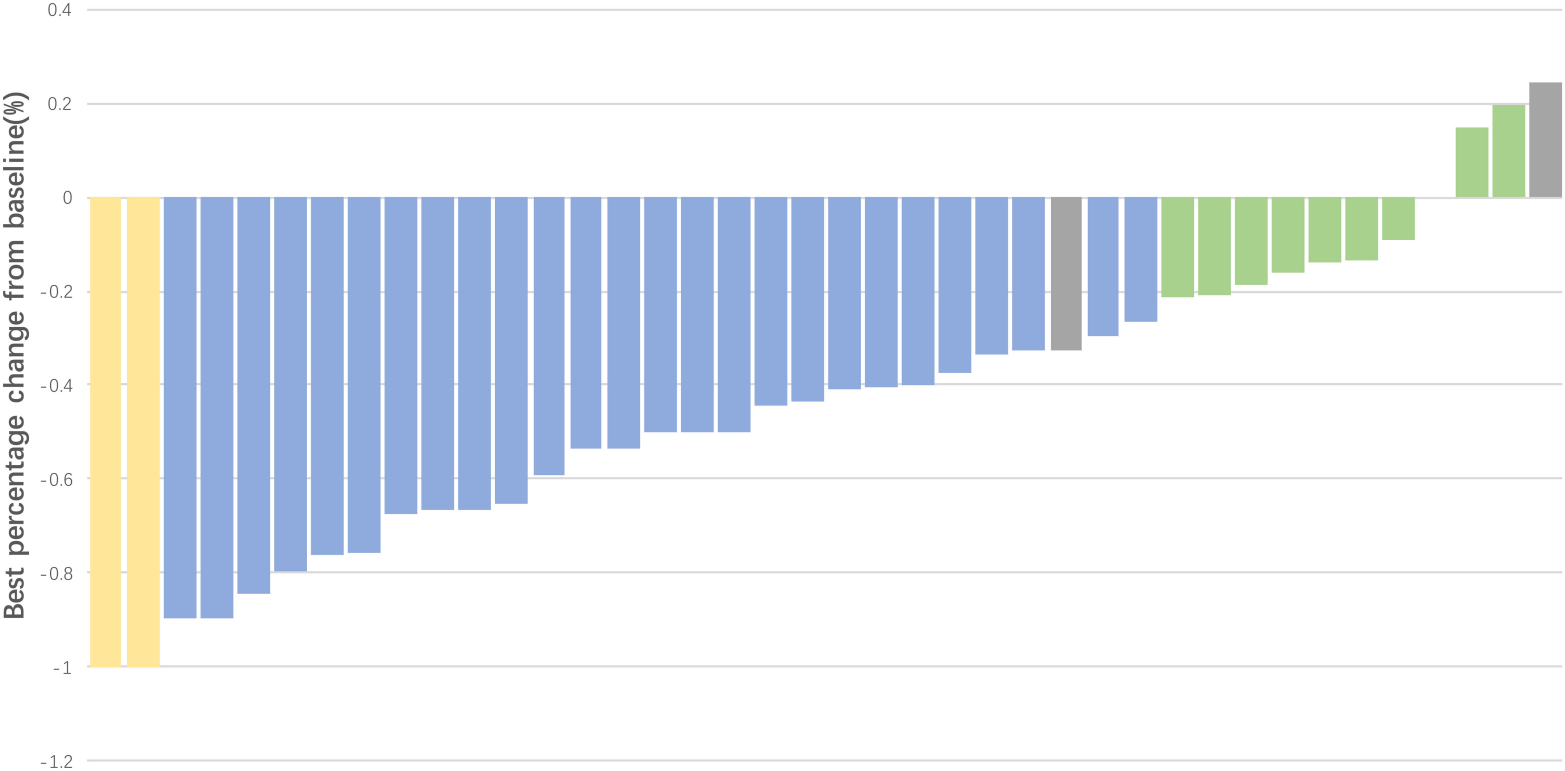
Waterfall plot for best percent change in lesion size from baseline in the efficacy analysis set (N = 40). CR, complete response (2 patients); PR, partial response (25 patients); SD, stable disease (11 patients); PD, progressive disease (2 patients). One patient was evaluated as PD due to the appearance of new lesions despite shrinkage of the original tumor.

**Figure 3:**
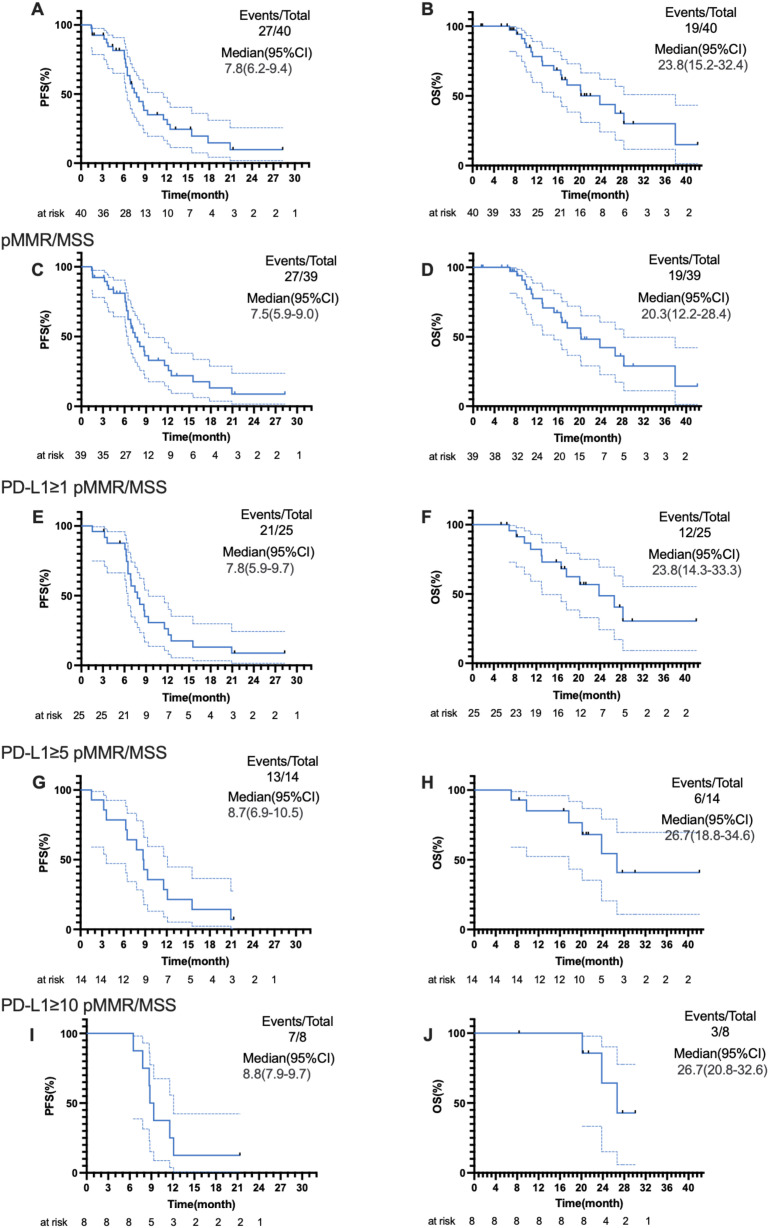
Kaplan-Meier curves for PFS and OS in the efficacy analysis set (N = 40) and subgroups. **(A, B)** PFS and OS for all patients (event maturity rates: 67.5% and 47.5%, respectively); **(C, D)** PFS and OS for patients with pMMR/MSS disease (event maturity rates: 69.2% and 48.7%, respectively); **(E, F)** PFS and OS for patients with pMMR/MSS disease and PD-L1 ≥1 (event maturity rates: 84% and 48%, respectively); **(G, H)** PFS and OS for patients with pMMR/MSS disease and PD-L1 ≥5 (event maturity rates: 92.9% and 42.9%, respectively); **(I, J)** PFS and OS for patients with pMMR/MSS disease and PD-L1 ≥10 (event maturity rates: 87.5% and 37.5%, respectively). PFS, progression-free survival; OS, overall survival; pMMR, mismatch repair-proficient; MSS, microsatellite-stable; PD-L1, programmed cell death-ligand 1.

In the first stage of this study, 13 of 15 patients achieved CR or PR, and the study successfully proceeded to the second stage. Among 40 patients in the efficacy analysis set, two achieved CR and 25 achieved PR (**Figure 2**), with an ORR of 67.5% (95% CI: 52.3%-82.7%). Eleven patients achieved SD, and the DCR was 95.0%. Median PFS was 7.8 (95% CI: 6.2-9.4) months, and median OS was 23.8 (95% CI: 15.2-32.4; **Figures 3A, B**) months.

For 39 patients with pMMR/MSS disease in the efficacy analysis set, the ORR was 66.7% (95% CI: 51.2%-82.1%), and the DCR was 94.9%. Median PFS was 7.5 (95% CI: 5.9-9.0) months, and median OS was 20.3 (95% CI: 12.2-28.4; **Figures 3C, D**) months. Further subgroup analyses showed that the median PFS and OS were 7.8 (95% CI: 5.9-9.7) months and 23.8 (95% CI: 14.3-33.3) months for 25 patients with pMMR/MSS disease and PD-L1 ≥1 (**Figures 3E, F**), 8.7 (95% CI: 6.9-10.5) months and 26.7 (95% CI: 18.8-34.6) months for 14 patients with pMMR/MSS disease and PD-L1 ≥5 (**Figures 3G, H**), and 8.8 (95% CI: 7.9-9.7) months and 26.7 (95% CI: 20.8-32.6) months for eight patients with pMMR/MSS disease and PD-L1 ≥10 (**Figures 3I, J**), respectively.

The univariate Cox regression analysis showed no associations of baseline characteristics with PFS or OS, except that sex was potentially associated with PFS (**Table 2**).

**Table 2:** Univariate analysis of associated factors for progression-free survival and overall survival.

Variable	Progression-free survival		Overall survival	
	HR (95%CI)	P	HR (95%CI)	P
Sex (female vs male)	0.281 (0.095-0.831)	0.022	0.525 (0.151-1.823)	0.311
Age	0.997 (0.955-1.040)	0.874	0.974 (0.932-1.018)	0.241
ECOG performance status				
0	Reference		Reference	
1	1.452 (0.596-3.536)	0.411	1.176 (0.406-3.409)	0.765
2	0.609 (0.207-1.795)	0.369	1.076 (0.323-3.585)	0.905
Histology (adenocarcinoma vs Signet-ring cell carcinoma)	1.558 (0.591-4.106)	0.370	0.797 (0.282-2.252)	0.669
HER2 status				
IHC 0	Reference		Reference	
IHC 1+	0.824 (0.431-1.576)	0.559	0.874 (0.380-2.010)	0.751
IHC 2+/FISH-	1.266 (0.690-2.324)	0.446	0.853 (0.373-1.954)	0.708
Peritoneal metastases (no vs yes)	1.445 (0.576-3.624)	0.433	2.251 (0.838-6.044)	0.107
Bone metastases (no vs yes)	0.854 (0.319-2.289)	0.754	0.651 (0.231-1.838)	0.418
Number of metastatic sites	1.264 (0.902-1.771)	0.174	1.029 (0.653-1.621)	0.901

ECOG, Eastern Cooperative Oncology Group; HER2, human epidermal growth factor receptor 2; IHC, immunohistochemistry; FISH, fluorescence *in situ* hybridization.

### Safety

Among 47 patients in the safety analysis set, 36 (76.6%) experienced TRAEs. The most common TRAEs were decreased neutrophil count (19 [40.4%]), hand-foot numbness (16 [34.0%]), anemia (11 [23.4%]), reactive cutaneous capillary endothelial proliferation (11 [23.4%]), and hypothyroidism (7 [14.9%]). 18 (38.3%) patients had grade 3–4 TRAEs, including decreased neutrophil count (12 [25.5%]), anemia (4[8.5%]), and immune-mediated rash (2 [4.3%]; **Table 3**). No severe immune-related inflammation such as immune-related pneumonia, hepatitis, or myocarditis occurred. The immune-related rash improved after subsequent treatment with glucocorticoid drugs, and the medication was not discontinued during this period. For hypothyroidism, oral thyroid hormone supplementation was administered. No treatment-related deaths occurred. Treatment-related information for patients with an ECOG performance status of 2 is shown in **Table 4**. Among these 7 patients, no serious adverse reactions occurred during treatment.

**Table 3:** Treatment-related adverse events.

Event	Patients (N = 47)	
	All grades (n, 95%CI) (%)	Grade 3-4 (n 95%CI) (%)
Anemia	11 (23.4, 12.3-38.0)	4 (8.5, 2.4-20.4)
Neutrophil count decreased	19 (40.4, 26.4-55.7)	12 (25.5, 13.9-40.3)
Platelet count decreased	2 (4.3, 0.5-14.5)	0(0)
Nausea/vomiting	6 (12.8, 4.8-25.7)	0(0)
Hand-foot numbness	16 (34.0, 20.9-49.3)	0(0)
Immune-mediated enteritis	1 (2.1, 0.1-10.9)	0(0)
Immune-mediated rash	2 (4.3, 0.5-14.5)	2 (4.3, 0.5-14.5)
Reactive cutaneous capillary endothelial proliferation	11 (23.4, 12.3-38.0)	0(0)
Creatinine increased	1 (2.1, 0.1-10.9)	0(0)
Creatine kinase increased	1 (2.1, 0.1-10.9)	0(0)
Hypothyroidism	7 (14.9,6.2-28.3)	0(0)
Hyperthyroidism	2 (4.3, 0.5-14.5)	0(0)
Liver function abnormality	1 (2.1, 0.1-10.9)	0(0)
Immune- mediated hepatitis, pneumonia, myocarditis, etc	0(0)	0(0)

Data are n (%).

**Table 4:** Treatment-related information for patients with an ECOG score of 2.

ID	Age	Sex	Number of treatment cycles	Adverse events	Metastatic sites
1	58	Female	34	Grade 2 thrombocytopenia, grade 2 granulocytopenia, hypothyroidism	Peritoneum metastasis
2	71	Female	21	Grade 1 nausea and vomiting	Peritoneum metastasis
3	71	Male	1	Grade 1 nausea and vomiting	Peritoneum metastasis, distant lymph node metastasis
4	53	Male	2	Grade 1 granulocytopenia	Lung metastasis, abdominal wall metastasis
5	50	Female	8	Grade 3 leukopenia, hypothyroidism	Pelvic metastasis, peritoneal metastasis
6	53	Male	1	Grade 1 granulocytopenia	Bone metastasis
7	75	Male	2	Grade 1 nausea and vomiting	Peritoneum metastasis

ECOG, Eastern Cooperative Oncology Group.

## Discussion

ICIs have ushered in a new era for the first-line treatment of HER2-negative advanced G/GEJ adenocarcinoma. Numerous clinical trials have demonstrated that combining immunotherapy with chemotherapy yields significantly greater benefits compared to chemotherapy alone. These pivotal trials of first-line immunochemotherapy predominantly used chemotherapy regimens incorporating fluoropyrimidine and platinum, resulting in a median PFS of 6.9-7.8 months (4–7), a median OS of 12.9-18.4 months (4–8, 28), and an ORR of 51.3%-70.6% (4–7, 28). When we designed this study, PD-1 monoclonal antibody inhibitors had not yet been approved in clinical guidelines for gastric cancer. A referenced meta-analysis showed that, compared with S-1 or taxane-based regimens as first-line treatment, platinum-containing regimens did not significantly improve efficacy or overall survival (OS); but the incidence of adverse events such as hematological toxicity, nausea, vomiting, and neurotoxicity was significantly higher (26). A phase II study also confirmed that S-1 combined with nab-paclitaxel (Nab-PTX) is an effective and safe regimen, which can be used as first-line treatment for patients with advanced gastric cancer (AGC) (29). Meanwhile, considering the continuous recommendation of taxane-based drugs in European and American guidelines, we chose to combine taxane-based drugs with S-1 and PD-1 monoclonal antibody inhibitors, aiming to provide more options and possibilities for clinical regimens.

The nanoscale size and excellent biocompatibility of albumin-bound paclitaxel facilitate rapid uptake by both tumor and immune cells. Moreover, internalized albumin-bound paclitaxel exhibits significant immunostimulatory activity, promoting the cancer immunity cycle. This activity includes enhanced tumor antigen presentation, T-cell activation, reversal of the immunosuppressive mechanisms within the tumor microenvironment, and synergistic interactions with cytotoxic lymphocytes to eliminate tumor cells (12, 24, 30). These findings suggest that albumin-bound paclitaxel can exert complementary effects when combined with immunotherapy. Based on this rationale, we evaluated the efficacy of combining albumin-bound paclitaxel and S-1 with camrelizumab for the first-line treatment of G/GEJ adenocarcinoma. In our study, the ORR was 67.5%, median PFS was 7.8 months, and median OS was 23.8 months. After excluding one patient with mismatch repair-deficient (dMMR) disease, the ORR was 66.7%, median PFS was 7.5 months, and median OS was 20.3 months. While ORR and PFS were comparable to those reported in previous studies (4–7, 28), the median OS demonstrated a remarkable improvement compared to other trials (4–8, 28). We believe the significant prolongation of overall survival (OS) may be attributed to the following factors: first, the patients had high compliance, along with meticulous clinical management and adequate supportive care; second, due to the small sample size, the study population may have had selection bias. In addition, the relatively short follow-up period, which leads to immature data, is also a limitation. We will continue to conduct and follow up on the subsequent follow-up work. Notably, our study enrolled patients with ECOG performance status of 2, and the proportion (14.9%) was significantly higher than that in CheckMate 649 (<1%) (4). Among patients with an ECOG performance status of 2, 2 patients failed to return to the hospital for treatment due to regional restrictions imposed by COVID-19, resulting in the lack of subsequent imaging data. For the remaining 5 patients, no serious adverse reactions occurred during the treatment period (**Table 4**). Additionally, other pivotal trials such as KEYNOTE-859, ORIENT-16, GEMSTONE-303, and RATIONE-305 excluded patients with ECOG performance status of 2. These findings suggest that the combination of camrelizumab, albumin-bound paclitaxel, and S-1 offers better tolerability. Gastrointestinal reactions are among the most easily perceived adverse events by patients, directly influencing treatment adherence. In our study, gastrointestinal reactions occurred in 12.8% of patients, and no grade 3 or 4 gastrointestinal reactions were observed. In contrast, regimens combining fluoropyrimidines with platinum consistently reported grade ≥3 gastrointestinal reactions, with an overall incidence exceeding 20% [CheckMate 649: 22%-38% (4); ORIENT-16: 37.5%-42.1% (7); RATIONE-305: 32%-48% (8)]. This suggests that camrelizumab combined with albumin-bound paclitaxel and S-1 results in reduced risk of gastrointestinal reactions, offering hope and alternative treatment options for patients with poor physical status.

In this study, Univariate analysis suggested a correlation between progression-free survival (PFS) and gender. However, this finding differs from that of comparative studies and other relevant research, which may be an accidental correlation caused by the small sample size. Due to the design of the preliminary experiment, one patient with deficient mismatch repair (dMMR) was enrolled. Nevertheless, given the small number of such cases, we believe it does not have a significant impact on the study results.

Subgroup analyses were conducted based on PD-L1 expression levels. Median PFS and OS were 7.8 months and 23.8 months for patients with pMMR/MSS disease and PD-L1 ≥1, 8.7 months and 26.7 months for patients with pMMR/MSS disease and PD-L1 ≥5, and 8.8 months and 26.7 months for patients with pMMR/MSS disease and PD-L1 ≥10, respectively. Although patients with PD-L1 ≥5 demonstrated substantial survival benefits compared to the all patients with pMMR/MSI disease, the incremental improvement in PFS for patients with PD-L1 ≥10 compared to those with PD-L1 ≥5 was minimal. Some pivotal trials reported survival results in patients with PD-L1 ≥5, with a median OS of 14.4 months in CheckMate 649 (4), a median PFS of 7.6 months and a median OS of 15.6 months in GEMSTONE-303 (6), and a median PFS of 7.7 months and a median OS of 18.4 months in ORIENT-16 (7). Our study results were better than those from these previous trials. We must also acknowledge that due to the small sample size, multivariate analysis could not be performed, which led to an overly wide confidence interval (CI) and potential overfitting. This subgroup analysis only serves as a *post-hoc* analysis to identify a trend. Current research suggests that the level of PD-L1 has limited value in predicting the efficacy of chemoimmunotherapy combinations. Further phase III studies will be needed in the future to validate this finding. Tissue sample collection was not conducted in our study; in the future, tissue sample collection and basic research validation can be carried out to further explore the impact of changes in the tumor microenvironment (TME) before and after treatment on prognosis.

Despite including patients with ECOG performance status of 2 in our study, the overall incidence of grade 3–4 TRAEs was not high (38.3%). This rate is notably lower compared to other pivotal trials, which reported grade 3–4 TRAE rate ranging from 53% to 60% (4, 5, 7, 8). In terms of specific adverse events, myelosuppression, hand-foot numbness, and gastrointestinal reactions were mainly related to chemotherapy. Reactive cutaneous capillary endothelial proliferation, thyroid dysfunction (hypothyroidism and hyperthyroidism), rash, and enteritis were mainly related to immunotherapy, consistent with previous reports (31). The incidence of chemotherapy-related adverse events in this study was not higher than that reported in studies utilizing fluoropyrimidine combined with platinum.

This study has several limitations. First, it was a single-arm, single-center study without a control group, which may have introduced selection bias. Second, the sample size was relatively small and the follow-up duration was short. Third, there were missing data on Lauren classification, no central radiological review, and no quality of life (QoL) assessment scale was established. Fourth, due to the excessively small sample size, multivariate analysis could not be conducted, and only exploratory univariate analysis was feasible. Additionally, the prevalence of the novel coronavirus (COVID-19) led to regional restrictions, which resulted in 7 patients failing to return to the hospital for continued treatment and thus not completing at least one radiological assessment. This situation also caused partial loss to follow-up and an excessively prolonged overall enrollment period. Identifying the population that can benefit more from immunochemotherapy remains an unresolved issue that requires further investigation.

In conclusion, the combination of camrelizumab, albumin-bound paclitaxel, and S-1 as first-line treatment for patients with HER2-negative advanced G/GEJ adenocarcinoma showed promising efficacy and an acceptable safety profile. Randomized controlled clinical trials are required to further demonstrate its efficacy and optimal application scenario.

## Data Availability

The raw data supporting the conclusions of this article will be made available by the authors, without undue reservation.
